# Exon arrays provide accurate assessments of gene expression

**DOI:** 10.1186/gb-2007-8-5-r82

**Published:** 2007-05-15

**Authors:** Karen Kapur, Yi Xing, Zhengqing Ouyang, Wing Hung Wong

**Affiliations:** 1 Department of Statistics, Stanford University, Stanford, California, 94305, USA; 2 Department of Internal Medicine, Roy J and Lucille A Carver College of Medicine, University of Iowa, Iowa City, Iowa, 52242, USA; 3 Department of Biological Sciences, Stanford University, Stanford, California, 94305, USA

## Abstract

We have developed a strategy for estimating gene expression on Affymetrix Exon arrays. The method includes a probe-specific background correction and a probe selection strategy in which a subset of probes with highly correlated intensities across multiple samples are chosen to summarize gene expression. Our results demonstrate that the proposed background model offers improvements over the default Affymetrix background correction and that Exon arrays may provide more accurate measurements of gene expression than traditional 3' arrays.

## Abstract 2

A strategy for estimating gene expression on Affymetrix exon arrays suggests that these arrays may provide more accurate measurements of gene expression than traditional 3’ arrays.

## Background

Microarray technology is a widely used high-throughput tool for measuring gene expression [1-3]. One of the most popular platforms is the Affymetrix GeneChip microarray. In this platform, gene-level expression indices are computed based on hybridization intensity measurements from multiple perfect match (PM) and mismatch (MM) probes targeting the 3' end of the mRNA sequence. The current generation of such 3' expression arrays includes the Human U133 Plus 2.0 array and the Mouse 430 2.0 array.

**Figure 1:**
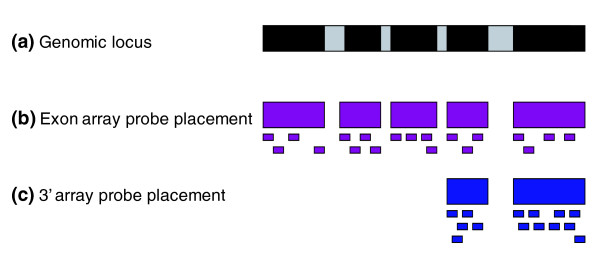
Probe design of Exon arrays. **(a) **Exon-intron structureof a gene. Black boxes represent exons. Gray boxes represent introns. Introns are not drawn to scale. **(b) **Probe design of Exon arrays. Four probes target each putative exon. **(c) **Probe design of 3' expression arrays. Probes target the 3' end of the mRNA sequence.

Recently, Affymetrix released a new platform, Exon arrays, designed to interrogate exon-level expression. Exon arrays differ significantly from 3' expression arrays in the number and placement of the oligonucleotide probes and in the design of control probes for background correction (Figure 1, modified from Affymetrix Exon Array design datasheet [4]). On Exon arrays, up to four probes are selected from each putative exonic region. In addition to probes targeting each exon supported by RefSeq mRNA evidence (core probes), Exon arrays also have probes that target exons supported solely by expressed sequence tag evidence (extended probes) or by purely computational predictions (full probes). In contrast to the 3' approach of using MM probes to measure non-specific hybridization, Exon arrays have no MM probes and instead include a set of probes designed to detect hybridization due to pure background. In all, there are over 6.5 million probes on the Human Exon 1.0 array, which represents an over four-fold increase in probe density and an eight-fold increase in the number of perfect-match targets compared to the Human U133 Plus 2.0 array. Can this dramatic increase in coverage be exploited to improve quantitative estimation of gene-level expression?

In this paper, we propose a strategy for computing gene expression indices on Exon arrays, called GeneBASE (Gene-level Background Adjusted Selected probe Expression). We employ a probe-specific background correction motivated by several recent studies using model-based approaches on 3' arrays [5] and on tiling arrays [6]. We combine a background correction model with our recently developed probe selection strategy [7] to construct a gene-level expression index for each transcript region. Although the ultimate goal of the new Exon array design is to provide exon-level expression information, the construction of a gene-level expression index is an important first task as it will provide a baseline against which to judge the expression of individual exons. To address the question of whether Exon arrays can provide improved gene-level expression measurements, we conducted a systematic comparison of Exon arrays and 3' arrays based on SAGE data. The results suggest that Exon arrays offer improved sensitivity and specificity of absence/presence (A/P) calls, and may allow more accurate quantitative measurement of the level of gene expression.

## Results

### Strategy for estimation of gene-level expression

Exon arrays differ from traditional 3' arrays in the approach to modeling non-specific hybridization. Exon arrays contain a set of 37,687 genomic and antigenomic background probes that are not matched to any putative transcript region. By default, the Affymetrix software estimates probe background signal (that is, its response to non-specific hybridization to off-target sequences) by the median response of all background probes with matching GC content to the probe in question. This background signal is then subtracted from the probe intensity to yield a background-corrected intensity. To explore whether it is possible to improve this background correction, we adapted a statistical model recently developed for Affymetrix genome tiling arrays [6] to predict background signal on Exon arrays. The model is a simple linear model of the log-probe-intensity that incorporates position specific nucleotide indicators and quadratic terms for nucleotide counts of the probe sequence (see Materials and methods for details). This model is referred to as the MAT model, named after the tiling array software in [6].

We used the Affymetrix Exon array tissue panel dataset, consisting of 11 human, tissues to evaluate the fit of the model trained from various sets of probes. It is important to train the MAT model using a set of probes detecting pure background signal. The set of background probes is ideal for fitting background models. We noticed that the set of full probes, which target exonic regions supported purely by computational predictions, tend to have low signals. We tested whether the set of full probes is appropriate for training background models. We also trained the MAT model using the set of core probes, which correspond to exonic regions supported by RefSeq mRNA evidence. The models trained on core probes are obtained just for comparison but not for use since core probes are likely to detect signal from their target transcripts and, therefore, would be inappropriate for training a background model. Training the MAT model using full or extended probes resulted in R^2 ^values of 61% to 64% and 59% to 64%, respectively, similar to those generated from training on background probes (64% to 67%) (Table 1). In contrast, training the model using core probes resulted in poorer performance (46% to 55%). Our results demonstrate that reliable estimates of background model parameters can be obtained using either the small set of background probes or the set of full probes on the array.

**Table 1:** Training the MAT background model using different sets of probes

Train/Test	Cerebellum	Heart	Liver
**Train on background probes, test on background probes R^2^**			
Cerebellum		0.64	0.67
Heart	0.64		0.65
Liver	0.66	0.64	
			
**Train on full probes, test on background probes R^2^**			
Cerebellum		0.61	0.63
Heart	0.61		0.63
Liver	0.64	0.63	

MAT background model parameters were estimated from background probes or full probes. After parameters were trained on a given tissue, the model is evaluated on separate tissues by the R^2 ^statistic.

We compared the performance of the MAT background correction to the Affymetrix default background correction algorithm of GC-matching. The MAT model is used to predict background signal of the probe in the log-intensity scale. To enable a comparison with GC-matching, the predicted GC-matching value is converted to the log scale to explain the log background intensity of each probe. Table 2 shows the proportion of variation (R^2^) in the set of full probes explained by the MAT model and the GC-matching. The results suggest that the MAT model is more effective, increasing the R^2 ^of the prediction of background probe response by 5% to 17%.

**Table 2:** Comparison of MAT and Affymetrix GC bin background models

	Cerebellum	Heart	Liver
**Train on background probes, test on full probes R^2^**			
MAT	0.24	0.30	0.35
GC Bin	0.07	0.24	0.25

The MAT and GC bin background models were trained from background probes. R^2 ^statistics are reported for the fit of background models to the set of full probes.

**Figure 2:**
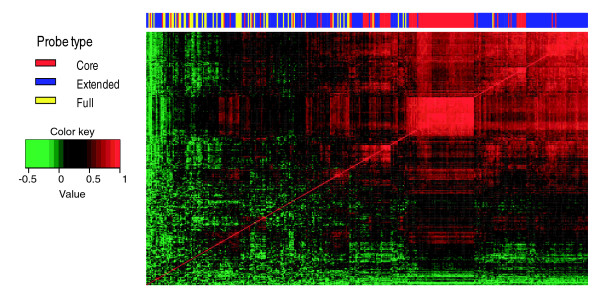
Heatmap visualization of Exon array pairwise probe correlations. Heatmap visualization of probe intensities of CD44 (Exon array transcript cluster 3326635). Each cell of the heatmap shows the correlation of two probe intensities among 11 tissues (breast, cerebellum, heart, kidney, liver, muscle, pancreas, prostate, spleen, testes, and thyroid). The top color bar indicates the probe annotation type, core probes (red), extended probes (blue), full probes (yellow). The signal intensities of core probes tend to have high correlation (the top right corner of the heatmap).

In addition to a more accurate background correction, gene expression estimates benefit from probe selection in which only reliable probes are used to estimate gene-level expression. For example, on 3' arrays, outlier detection removes poorly performing probes [8]. Compared to 3' arrays, there is a substantial increase in the number of probes per gene on Exon arrays, motivating a stringent probe selection procedure. Although probes are chosen to be specific to their corresponding target sequence, not all probes perform well. Some probes respond poorly to target signals, while other probes cross-hybridize to non-specific gene targets. Probes targeting alternatively spliced regions may also be inappropriate indicators of overall gene-level expression. We have recently developed an effective probe-selection algorithm [7]. Figure 2 illustrates the basic idea. First, background-corrected probe intensities are normalized using a simple normalization scheme in which a multiplicative scaling factor is applied so that the median intensity of all core probes in an array equals 100. Using the Affymetrix human tissue panel dataset, we created a correlation heatmap to select a set of highly correlated probes. As shown in Figure 2, the set of core probes, which target exonic regions supported by RefSeq mRNA evidence, tends to have highly correlated intensities. However, it is apparent that not all core probes are reliable indicators of gene expression. Our probe selection strategy selects a set of probes with reliable signal intensities, improving gene expression estimation. More details on probe selection can be found in Materials and methods.

After background correction, normalization and probe selection, we are ready to compute a gene-level expression index. We do this by adapting a model, first proposed in [8] for 3' arrays, to fit the gene expression index for each gene (see Materials and methods). This approach requires multiple arrays (four or more are recommended) and the gene expression indices are estimated more accurately when there is substantial variation in gene-level expression across the different arrays. We have found that the data from the Exon array platform is sufficiently uniform after normalization so that arrays from different studies may be combined for model fitting. Thus, for small scale studies with a limited number of arrays, we recommend combining the arrays with the Affymetrix tissue panel arrays to compute the expression index.

Finally, in any experiment, a substantial number of genes may be expressed at such a low level that they are undetectable by the array. It is often desirable to remove such 'absent' genes from further quantitative analyses [9]. To identify the absent genes, we compare the observed probe intensities to their background levels predicted by the MAT model. A gene is called 'absent' if the statistical test shows that the probe intensities are not significantly different from background (see Materials and methods).

### Evaluation of exon array gene-level expression measurements

We used existing tissue panel expression datasets to study the performance of Exon arrays and 3' arrays. The datasets consist of expression profiles from Human Exon 1.0 ST arrays and U133 Plus 2.0 arrays on 11 human tissues. For each array type, three replicates are available for each tissue. To evaluate the accuracy of A/P calls from Exon arrays as discussed above, we first construct gold-standard sets of 'present' genes and gold-standard sets of 'absent' genes using an independent data source. For each tissue type, these gold-standard sets are constructed based on an analysis of available SAGE libraries of the same tissue type (see Materials and methods).

**Figure 3:**
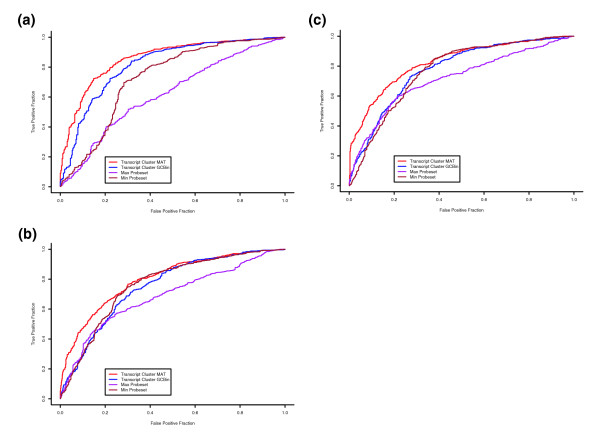
Comparison of A/P calls using ROC curves. Different models of probe-specific background are used as the basis for generating A/P calls of gene expression. We plot the ROC curve as the true positive rate versus the false positive rate of agreement between each A/P call method and gold-standard sets of expressed and unexpressed genes generated from independent SAGE data. The ROC curves from several A/P methods are shown here in each of three tissues, **(a) **cerebellum, **(b) **heart, **(c) **liver: Exon array MAT background (red); Exon array Affymetrix DABG (blue); maximum 3' array MAS 5.0 probeset statistic (purple); minimum 3' array MAS5.0 probeset statistic (brown).

We ranked genes belonging to the gold-standard present or absent sets that were jointly contained on both 3' and Exon arrays according to the *p *values for the A/P call in each array (see Materials and methods for details of A/P call methods). For a given ranking threshold value, genes ranked less than the threshold are called present and genes ranked greater than the threshold are called absent. Using the gold-standard genes from SAGE libraries as ground truth, we can estimate the true positive rate as well as the false positive rate of the A/P call method. As we increase the threshold, the true positive rate for detecting present genes increases but the false positive rate also increases. The range of specificity and sensitivity attainable by the Exon array is given by the receiver operating characteristic (ROC) curves in Figure 3. In the case where a gene on the Exon array corresponds to multiple probesets on the 3' array, we considered both the maximum or minimum *p *value. An ROC curve is plotted for each case, shown in Figure 3. It is clear from the figure that Exon array MAT A/P calls are substantially more accurate than 3' array A/P calls. For example, for the first heart tissue sample, at a false positive rate of 10%, using the Exon array MAT background correction, we detected a true positive rate of 47% while the 3' array detected a true positive rate of only 32%.

**Figure 4:**
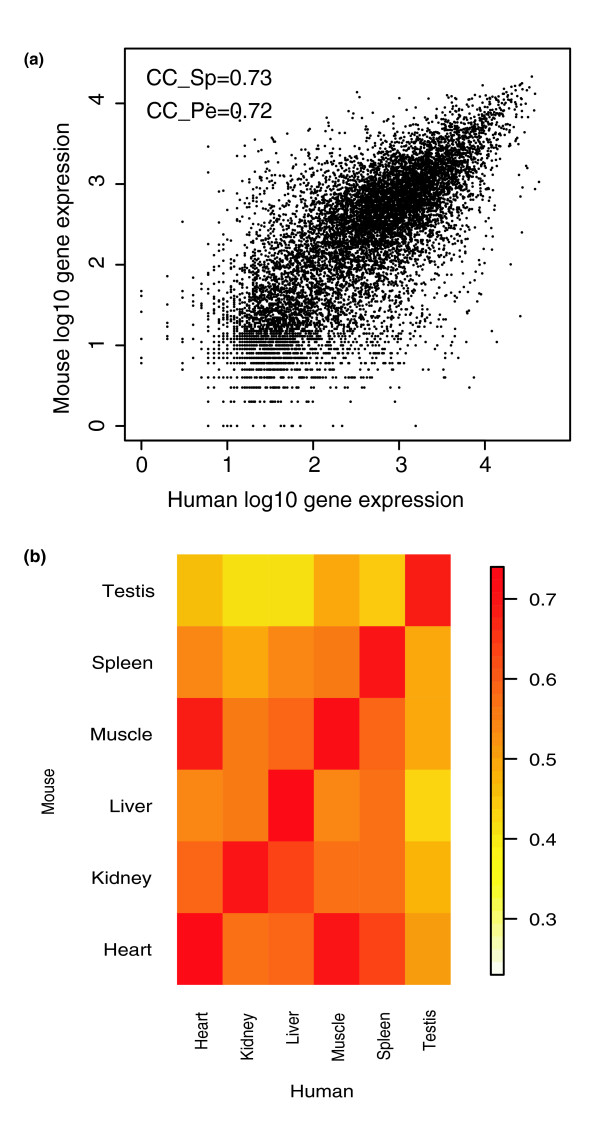
Correlation of expression indices between human and mouse. Gene expression indices on a set of ortholog genes were computed using identical human and mouse tissues from Exon arrays. **(a) **Exon array ortholog gene correlations on heart tissue. **(b) **Exon array correlation of tissue expression between human and mouse.

Next, we sought to assess the accuracy of the quantitative gene-level expression indices provided by Exon arrays. Since it is difficult to establish quantitative ground truth on gene expression on a whole genome scale, we rely on an indirect assessment. We reason that if the expression index is accurate quantitatively, then one can expect to see a high degree of cross-species correlation of gene expression between ortholog genes. We established ortholog gene pairs between human and mouse based on NCBI's HomoloGene database. Using Exon array datasets from the Affymetrix human and mouse tissue panel study, for each tissue we computed the cross-species correlation between the human and mouse expression values across all ortholog gene pairs. The results are presented in Figure 4. The pattern of cross-species conservation of tissue-specific gene expression is evident. For example, in heart tissue the Spearman correlation between Exon array expression indices in human and mouse orthologs reached a level of 0.73, which is much higher than previously reported figures [10,11]. Furthermore, Exon arrays reveal patterns of cross-species gene expression. For example, in terms of gene expression, not only is human heart tissue similar to mouse heart tissue, but it is also similar to mouse skeletal muscle. The large degree of conservation between cross-species gene expression is an interesting biological result, indirectly supporting improved gene expression estimates on Exon arrays.

## Discussion

The recently released Exon arrays differ from the previous 3' arrays in terms of the number, placement and annotational confidence of oligonucleotide probes. As a result, new methods that take advantage of Exon array design features can improve gene-level expression estimates. In this manuscript we propose a strategy for computing gene expression indices on Exon arrays that combines a probe-specific background correction with a probe selection procedure. Analysis of independent SAGE data demonstrates that A/P calls generated from the MAT background model offer substantial improvements. This improvement is likely due to both the increased number of probes per gene and the improvement of the MAT background model over the default Affymetrix background correction. Furthermore, we observed that Exon array gene expression indices show a high degree of correlation between human and mouse orthologs.

This work represents a step in the continued development of accurate gene-level expression estimates from microarray data. However, other approaches to estimating gene-level expression are possible, including model-based approaches [8,12-14], and methods based on physical models [15,16]. Various methods for estimating probe background intensities have also been proposed, incorporating probe content [17,18] or using physical models [19]. With so many probes interrogating background signal, Exon arrays present the opportunity to fit improved background models. Extensions of the MAT model [6] may offer future improvements in estimating background intensities. Alternative methods for probe selection are also possible. For example, Affymetrix proposes an iterative probe selection strategy, IterPLIER [20], in which a subset of probes of fixed size (11 probes) is iteratively chosen.

Accurate gene-level expression estimates are useful not only for high-level analysis but also serve as a first step for detecting alternative splicing [21-25]. For example, to detect alternative splicing we can track the exon-level expression relative to overall gene expression. Accurate baseline gene expressions will enable more sensitive detection of alternative splicing.

## Conclusion

We describe a strategy for estimating gene-level expression indices on Exon arrays that incorporates a probe-specific background correction with a probe selection procedure. We validated our approach using independent SAGE data and cross-species comparisons.

## Materials and methods

### MAT background model

We applied the following background model, adapted from the MAT model on tiling arrays [6], to Exon arrays:

logBkg(PMi)=αniT+∑j=125∑   k∈{A,C,G}βjkIijk+∑k∈{A,C,G,T}γknik2+εi
 MathType@MTEF@5@5@+=feaafiart1ev1aaatCvAUfeBSjuyZL2yd9gzLbvyNv2Caerbhv2BYDwAHbqedmvETj2BSbqee0evGueE0jxyaibaiKI8=vI8tuQ8FMI8Gi=hEeeu0xXdbba9frFj0=OqFfea0dXdd9vqai=hGuQ8kuc9pgc9s8qqaq=dirpe0xb9q8qiLsFr0=vr0=vr0dc8meaabaqaciGacaGaaeqabaqadeqadaaakeaatCvAUfKttLearyWrP9MDH5MBPbIqV92AaGabaiab=XgaSjab=9gaVjab=DgaNjaadkeacaWGRbGaam4zaiaacIcacaWGqbGaamytamaaBaaaleaacaWGPbaabeaakiaacMcacqGH9aqpiiGacqGFXoqycaWGUbWaaSbaaSqaaiaadMgacaWGubaabeaakiabgUcaRmaaqahabaWaaabuaeaacqGFYoGydaWgaaWcbaGaamOAaiaadUgaaeqaaOGaamysamaaBaaaleaacaWGPbGaamOAaiaadUgaaeqaaaqaaiaadUgacqGHiiIZcaGG7bGaamyqaiaacYcacaWGdbGaaiilaiaadEeacaGG9baabeqdcqGHris5aaWcbaGaamOAaiabg2da9iaaigdaaeaacaaIYaGaaGynaaqdcqGHris5aOGaey4kaSYaaabuaeaacqGFZoWzdaWgaaWcbaGaam4AaaqabaGccaWGUbWaa0baaSqaaiaadMgacaWGRbaabaGaaGOmaaaakiabgUcaRiab+v7aLnaaBaaaleaacaWGPbaabeaaaeaacaWGRbGaeyicI4Saai4EaiaadgeacaGGSaGaam4qaiaacYcacaWGhbGaaiilaiaadsfacaGG9baabeqdcqGHris5aaaa@7B06@

where *PM*_*i *_is the intensity of the perfect match probe *i*, *n*_*ik *_is the count of nucleotides of type *k *in the probe sequence, *I*_*ijk *_is the indicator of nucleotide of type *k *in position *j*, *α*, *β*_*jk*_, *γ*_*k *_are parameters in the model, and *ε*_*i *_is a probe-specific error term. Parameters were estimated by least squares using a set of probe intensities believed to be detecting mostly background. In this paper MAT parameters were fit using either the set of background probes or the set of full probes on Exon arrays.

### R^2 ^model fit statistic

We compared the effect of training the MAT background model using different sets of probes. The MAT parameters were estimated using the given set of probes on one array. On a separate array, a single scale parameter was fit to adjust for overall background abundance. The R^2 ^statistic was reported for the set of estimated background probe intensities on the separate array.

We compared the MAT background model with the Affymetrix GC bin background correction by computing an R^2 ^statistic as follows. Each background model was trained using the background probes on a given array. On the same array, we computed the R^2 ^statistic from the estimated intensities of full probes. The R^2 ^statistic is given in terms of the log probe intensities.

### Tissue panel dataset

We downloaded the public Human and Mouse Exon 1.0 ST Array and U133Plus2.0 Array tissue panel dataset [26] consisting of 11 tissues (breast, cerebellum, heart, kidney, liver, muscle, pancreas, prostate, spleen, testes, and thyroid), each with three replicates.

### Probe selection

Probe selection was performed using the Affymetrix tissue panel dataset. The details of the probe selection algorithm have been detailed previously [7].

### Exon array gene-level expression estimation

Background corrected, normalized, selected probe intensities were used to summarize gene expression using the model described in [8].

### Mapping genes between 3' arrays and exon arrays

We used the mapping file provided by Affymetrix [27] to match genes between the U133 Plus 2.0 array and the Human Exon array. We required that a gene had at least one core probe on the exon array. The filtering resulted in a set of 17,165 genes mapped between the two arrays.

### Absence/presence calls

For the 3' arrays, the Affymetrix MAS 5.0 algorithm [28] uses the PM and MM probes to calculate a discrimination score for each probe pair:

R = (PM - MM)/(PM + MM)

First, the algorithm tests whether the PM and MM probe pairs are saturated. If all probe pairs are saturated, then the probeset is automatically declared present. Otherwise, the set of discrimination scores corresponding to probe pairs within a probeset are used to carry out a one-sided Wilcoxon's signed rank test, with null hypothesis parameter τ = 0.015 as the default value. Each probeset is assigned a detection *p *value used to make A/P calls.

For the Exon arrays, Affymetrix implements the detection above background (DABG) method for making A/P calls. Each probe is assigned an empirical *p *value, obtained by comparing the probe intensity to the distribution of background probe intensities with identical GC content. The *p *values are transformed via the formula *x *= -2log(*p*). Under the null hypothesis that the probes are detecting background, the *p *values follow a uniform [0,1] distribution and the transformed *p *values follow a χ22
 MathType@MTEF@5@5@+=feaafiart1ev1aaatCvAUfeBSjuyZL2yd9gzLbvyNv2Caerbhv2BYDwAHbqedmvETj2BSbqee0evGueE0jxyaibaiKI8=vI8tuQ8FMI8Gi=hEeeu0xXdbba9frFj0=OqFfea0dXdd9vqai=hGuQ8kuc9pgc9s8qqaq=dirpe0xb9q8qiLsFr0=vr0=vr0dc8meaabaqaciGacaGaaeqabaqadeqadaaakeaacqaHhpWydaqhaaWcbaGaaGOmaaqaaiaaikdaaaaaaa@3689@ distribution. The sum of the transformed *p *values follows a χ2k2
 MathType@MTEF@5@5@+=feaafiart1ev1aaatCvAUfeBSjuyZL2yd9gzLbvyNv2Caerbhv2BYDwAHbqedmvETj2BSbqee0evGueE0jxyaibaiKI8=vI8tuQ8FMI8Gi=hEeeu0xXdbba9frFj0=OqFfea0dXdd9vqai=hGuQ8kuc9pgc9s8qqaq=dirpe0xb9q8qiLsFr0=vr0=vr0dc8meaabaqaciGacaGaaeqabaqadeqadaaakeaacqaHhpWydaqhaaWcbaGaaGOmaiaadUgaaeaacaaIYaaaaaaa@3779@ distribution, where k equals the number of probes.

We applied the MAT model to generate A/P calls. After fitting the MAT model, we use the linear model to compute an estimate of standard error for the predicted values. Each probe intensity is given a Z-score. The sum of the Z-scores is standardized to follow a standard Normal distribution.

### Gold-standard A/P genes from SAGE data

For each tissue, SAGE data were used to construct gold-standard expressed and unexpressed sets of genes. We used the NCBI SAGEmap tool [29] to select tags present in the Cancer Genome Anatomy Project (CGAP) SAGE normal tissue libraries (GEO accession: GSE14). Eight of the eleven tissues surveyed in the tissue panel dataset had corresponding libraries (breast, cerebellum, heart, kidney, liver, pancreas, prostate, and thyroid). SAGE tags were mapped to the most likely corresponding UniGene cluster, which was subsequently mapped to genes on the U133 Plus 2.0 and Exon arrays. A gene was declared to belong to the reference set of expressed genes if it had a value of at least 100 tags per million (tpm). A gene was declared to belong to the reference set of unexpressed genes if: it had no SAGE tags observed in the given tissue; and it was expressed (that is, tpm ≥ 100) in at least one other SAGE tissue library.

### Absence/presence ROC curves

ROC curves were constructed to compare the performance of the different methods of A/P calls. Genes were ranked by the *p *values computed from different A/P methods. The ROC curves were constructed using the threshold of gene rank. Specifically, for each value of k from 1 to N, where N equals the number of common genes between 3' and Exon arrays, the top k genes and the bottom N - k genes from each A/P call method were compared to the gold standard sets of expressed and unexpressed genes constructed from SAGE libraries. We used the reference sets of known positives and known negatives to count the numbers of true positives (TP), false positives (FP), true negatives (TN) and false negatives (FN). We calculated the true positive rate and false positive rate at a particular gene number cutoff as:

TP%=TPTP+FN
 MathType@MTEF@5@5@+=feaafiart1ev1aaatCvAUfeBSjuyZL2yd9gzLbvyNv2Caerbhv2BYDwAHbqedmvETj2BSbqee0evGueE0jxyaibaiKI8=vI8tuQ8FMI8Gi=hEeeu0xXdbba9frFj0=OqFfea0dXdd9vqai=hGuQ8kuc9pgc9s8qqaq=dirpe0xb9q8qiLsFr0=vr0=vr0dc8meaabaqaciGacaGaaeqabaqadeqadaaakeaacaWGubGaamiuaiaacwcacqGH9aqpdaWcaaqaaiaadsfacaWGqbaabaGaamivaiaadcfacqGHRaWkcaWGgbGaamOtaaaaaaa@3C76@

FP%=FPFP+TN
 MathType@MTEF@5@5@+=feaafiart1ev1aaatCvAUfeBSjuyZL2yd9gzLbvyNv2Caerbhv2BYDwAHbqedmvETj2BSbqee0evGueE0jxyaibaiKI8=vI8tuQ8FMI8Gi=hEeeu0xXdbba9frFj0=OqFfea0dXdd9vqai=hGuQ8kuc9pgc9s8qqaq=dirpe0xb9q8qiLsFr0=vr0=vr0dc8meaabaqaciGacaGaaeqabaqadeqadaaakeaacaWGgbGaamiuaiaacwcacqGH9aqpdaWcaaqaaiaadAeacaWGqbaabaGaamOraiaadcfacqGHRaWkcaWGubGaamOtaaaaaaa@3C5A@

### Cross-species comparison

We downloaded the publicly available human and mouse tissue panel datasets from Affymetrix. Exon array gene expressions were computed using the background correction, scale normalization and probe selection procedure described here. For each tissue, gene-level expression indices were averaged over the three replicates.

Using NCBI's HomoloGene database [30], we extracted 16,044 non-redundant orthologous gene pairs between human and mouse. A total of 10,480 gene pairs were represented on human and mouse Exon arrays. For a given tissue, we computed the cross-species correlation as the Pearson or Spearman correlation computed over the set of ortholog gene pairs.

### Available software

We provide freely available software to compute gene expression indices on Exon arrays. We implement an additive MAT background correction and a simple normalization scheme where the background corrected intensities of core probes are scaled to have median intensity of 100. Additional background correction and normalization options are provided. See [31] for further details.
